# Transitioning to Aboriginal community control of primary health care: the process and strategies of one community-controlled health organisation in Queensland

**DOI:** 10.1186/s12875-020-01300-z

**Published:** 2020-11-10

**Authors:** Crystal Jongen, Sandra Campbell, Janya McCalman, Ruth Fagan, Kingsley Pearson, Suzanne Andrews

**Affiliations:** 1 School of Health, Medical and Applied Sciences, Central Queensland University, Cairns, Australia; 2 Centre for Indigenous Health Equity Research (CIHER), Central Queensland University, Cairns, Australia; 3 Gurriny Yealamucka Health Services Aboriginal Corporation, Yarrabah, Australia

**Keywords:** Aboriginal community control, Transition to community control, Primary health care, Self-determination, Health equity, Social and cultural determinants of health

## Abstract

**Background:**

Aboriginal Community Controlled Health Services (ACCHSs) play a critical role in providing culturally appropriate, accessible primary healthcare (PHC) for Aboriginal and Torres Strait Islander peoples in Australia. The success of many ACCHSs has led to increased policy support for their growth and development, including the transition of state government administered PHC services to Aboriginal community control in select communities. However, there is minimal published literature available which evaluates such transitions. This paper reports on an evaluation of one ACCHS (Gurriny Yealamucka Health Service)‘s experience of transitioning local PHC services to community control in Yarrabah, Queensland, with a focus on the processes and strategies which were implemented to achieve successful transition.

**Methods:**

Data was collected from interviews with key personnel involved in the transition and organisational documents from the evaluation period. Face-to-face or telephone interviews were conducted with 14 key stakeholders, audio-recorded and transcribed with written consent. Historical organisational documents were provided by Gurriny. All interview transcripts and documents were imported into NVIVO, coded and analysed using grounded theory methods.

**Results:**

Gurriny’s journey of achieving community control of PHC in Yarrabah entailed an almost 30 year process of building and demonstrating organisational capacity. The first stage (1986 to 2004) was focused on establishing and developing a community-controlled health service and the second stage (2005–14) on preparing for the transition. Formal handover occurred in June 2014. Stage one strategies included: addressing community social and emotional wellbeing; consulting the community; collaborating with researchers; and, strategically building services, organisation capacity and stakeholder trust. Stage two strategies were: communicating and engaging with stakeholders; ensuring strong governance; planning and developing the services and workforce; assuring quality; and, financial planning, management and modelling.

**Conclusion:**

Achieving successful transition to community control of PHC for Gurriny entailed a lengthy process of substantial, ongoing organisational growth and development. Gurriny’s experience provides a framework for both governments and the ACCHS sector to inform future transitions of PHC services to Aboriginal community control.

## Background

The United Nations Declaration on the Rights of Indigenous Peoples affirms the rights of Indigenous people globally to self-determination and autonomy in the pursuit of economic, social and cultural development [1]. This includes the “the right to maintain and strengthen their distinct political, legal, economic, social and cultural institutions” (p. 5) [1]. Indigenous community control of local primary health care (PHC) services is one key expression of this right to self-determination which can be seen across many countries [2]. Although operating under distinct legal, policy and contractual contexts [3] many Indigenous community controlled PHC services globally are directed by Indigenous governance structures and are designed to be culturally appropriate and responsive to local community health needs [4]. The success of Indigenous community control of PHC models has led to their endorsement and promotion by governments “as a mechanism to improve Indigenous participation in health care, increase access and reduce in equities.” (p.6) [4].

In Australia, the 140 Aboriginal Community Controlled Health Services (ACCHSs) operating comprise more than two-thirds (71%) of the PHC service sector delivering care to Aboriginal^1^ and Torres Strait Islander peoples [5]. ACCHSs are grounded in local values and culture, governed and operated by local communities [6] and provide healthcare based on Aboriginal concepts of holistic health and wellbeing for the whole community [7]. In this way ACCHSs work to improve the availability, affordability, cultural acceptability and appropriateness of PHC to Aboriginal people’s health needs’ [8, 9]. The positive impacts achieved by ACCHSs can be seen across healthcare, workforce and community outcomes. These include high access rates, superior performance on key best-practice PHC indicators, the provision of employment to large numbers of Aboriginal and Torres Strait Islander workers [9] as well as the facilitation of increased community participation, engagement, empowerment and control [10]. Given the importance of self-determination and control as key determinants of health for Aboriginal people [11], ACCHSs can be considered a health intervention in their own right [12].

The first ACCHSs were founded in the 1970s in the context of land rights and other social movements [13]. They were established in response to racism experienced by Aboriginal people in mainstream health services as well as their poor affordability, cultural acceptability and appropriateness to health needs [14]. This has resulted in ACCHSs largely being established as independent, community organisations in an ad hoc manner across Australia [15]. Early ACCHSs were initiated with little to no government support or funding, and even outright government opposition [16, 17]. However, since the 1980s, support for Aboriginal control of PHC has been expressed in various national and state-based government policies [18]. Since the mid-90s there has been policy support for the transition of government run PHC services to Aboriginal community control [15] leading to the successful transition of various PHC services to Aboriginal community control in the Northern Territory, Queensland and South Australia [10]. However, there is limited literature that documents such processes of transition, hence limited guidance for other services to undertake community control of PHC governance and operations.

In 2005, a Deed of Commitment was signed for the transition of Queensland Health PHC services delivered by the Cairns and Hinterland Hospital and Health Service (CHHHS) in Yarrabah to the community-controlled organisation, Gurriny Yealamucka Health Service Aboriginal Corporation (Gurriny). Queensland Health (QH), the federal Department of Health and Ageing, the Yarrabah Aboriginal Shire Council and Gurriny were co-signatories. Handover to community control of PHC by Gurriny was eventually operationalised nearly a decade later in June 2014. This paper emanates from an evaluation of the transition process and community, health, workforce and financial impacts contracted by QH in November 2017. This paper focuses on the core process underlying Gurriny’s transition to community control and the key strategies that were implemented to make it a success. It provides a framework of the Yarrabah case that can inform other ACCHSs and policy makers wishing to pursue transitions to community control of PHC by outlining what was done to achieve a successful transition in Yarrabah. Other results which emerged from the evaluation report on the barriers and enablers to, and outcomes and impacts of, the transition to Aboriginal community control of PHC in Yarrabah [19].

## Methods

### Community research partnership approach

The transition to community control evaluation project reported in this paper was contracted by QH and implemented as a partnership between Gurriny and the Centre for Indigenous Health Equity Research (CIHER) at Central Queensland University (CQU). A Research Services Agreement was created to formally establish the relationship between Gurriny and CIHER in the research collaboration. This agreement established Gurriny’s rights of access to and ownership of their existing intellectual and cultural property and intellectual and cultural property created through the research.

A Steering Committee, chaired and coordinated by Gurriny, was established to inform and guide the research. Members of the Steering Committee included representatives from the Aboriginal and Torres Strait Islander branch of QH, CHHHS, Queensland Aboriginal and Islander Health Council (QAIHC) and the research team. More than 50% of the Steering Committee members were Aboriginal and/or Torres Strait Islander. The Gurriny team (RF, KP, SA) managed Gurriny’s partnership in all stages of the project from identifying and setting the research priority through to translation and dissemination of findings. The CIHER team (SC, JM, CJ) iteratively provided the results of data analysis to the Steering Committee and Gurriny leaders for feedback and the opportunity to request changes to the analysis strategies and reporting of results. Approval was sought from Gurriny prior to publishing any evaluation findings.

The evaluation findings were presented to approximately 40 Gurriny staff at a Gurriny Senior Management Team meeting and Gurriny staff meeting in 2019. Positive feedback was received, with one staff member suggesting that information be included in Gurriny staff orientation processes. A final evaluation report [19] was provided to Gurriny who shared this document with QH stakeholders. Permission was also granted from Gurriny to share the final evaluation report with relevant stakeholders including a senior policy officer for Aboriginal health at the federal level, other senior Indigenous researchers, and other Aboriginal and Torres Strait Islander community-controlled health services.

### Data gathering and analysis

Interviews with key personnel involved in the transition to community control of PHC in Yarrabah, as well as Gurriny’s organisational documents from the evaluation period, were analysed using grounded theory methods. Ethics approval was received from Far North Queensland Human Research Ethics Committee of the Cairns and Hinterland Hospital and Health Service (CHHHS) and Central Queensland University.

#### Participant recruitment and sampling

A purposeful sampling technique was initially used to identify and select information-rich participants. To start the process, senior managers at Gurriny and QH identified individuals who they believed would be appropriate participants. These and self-identified participants were invited to contribute through interviews that focused on their experiences of the transition; including enablers, barriers, strategies and impacts.

As data collection progressed, the focus of interviews moved to explore emerging issues from the initial data. Participants were selected based on their roles in the transition and/or unique perspectives, with theoretical sampling processes being used to identify potential participants based on a diversity of perspective and their potential to provide information about the emergent theoretical issues. Table 1 provides a summary of requests to interview, and participation. Eight of the 14 people interviewed were Aboriginal.

**Table 1:** Requests and participation in interviews

Participant	Requested for interview	Interviewed
Ex Gurriny staff member	3	2
Current Gurriny staff member	9	3
Current Gurriny staff member who previously worked for QH		3
Other Yarrabah community member	3	1
Ex QH staff member	6	3
Current QH staff member	6	1
Other	1	1
TOTAL	29	14

Face-to-face or telephone interviews with current and ex Gurriny staff and Board members, Yarrabah community members, and current and ex QH staff were undertaken by SC and JM. A culturally sensitive engagement strategy was implemented whereby SC, an Aboriginal researcher, spent 1 day a week at Gurriny to build relationships with Gurriny staff, have informal conversations about the project and invite people to participate All potential participants were provided with a participant information sheet and informed of their right to not participate or to withdraw without prejudice. All signed a participant consent form. Interviews were audio-recorded and transcribed. Participants were invited to review, approve or correct their interview transcript prior to processing, and/or receive copies of project results. Interviews were conducted at a place of the participant’s choice. During data collection, flexibility was required to respect local community protocols. In instances when there was sorry business^2^ in the community or unexpected community events, data gathering or other research activities involving community members were put on hold.

#### Historical documents

Qualitative analysis of informative historical organisational documents (provided by Gurriny) as a means of ‘triangulation’ augmented the data arising from face-to-face stakeholder interviews. Documents analysed pertained to planning and implementation issues related to transition.

#### Data analysis

Data from the interviews and documents were analysed using the constant comparison methods of grounded theory. The interview data and documents were imported into NVIVO software and coded. Open-coding commenced upon receipt of the first transcripts using segment-by-segment coding [20]. Open-codes or concepts were generated by asking three questions: 1) What is really going on here relative to implementation of transition to community control? What concept is involved? and What is the basic problem faced by the participants? [21]. In axial coding, building the theory of implementation involved constantly comparing new data to existing concepts for similarities and differences; identifying any new concepts from subsequent transcripts; identifying the relationships between concepts; uncovering the dimensions of the concepts to explain how they were operationalised and demonstrating commonalities and variances; and continually verifying commensurate and dissenting interpretations of these concepts in additional data. Concepts that identified events, incidents, actions and interactions that were related in meaning were grouped under higher order concepts termed categories [22]. Selective coding involved integrating and refining the categories and their sub-categories into a theoretical framework that explained the transition process. The theoretical framework itself became a set of relational statements about the categories concerning what was happening in the implementation process [22].

## Results

### The core process: building and demonstrating Yarrabah’s capacity

The core process which emerged from the grounded theory analysis was one of building and demonstrating capacity. Gurriny’s journey of achieving community control of PHC in Yarrabah entailed an almost 30 year process of building and demonstrating community and organisational capacity. This process started small, using available resources to iteratively build programs, services and the organisation itself.

“Gurriny had to start small and work their way up.” (Ex Gurriny staff)

For example, a feasibility study undertaken in 1998 which articulated and strengthened the community vision for community control of healthcare, prompted a strategic decision to develop a social and emotional wellbeing (SEWB) focus and services. Over the following years, Gurriny established itself as a Centre of Excellence in SEWB, drawing from research partnerships to build community and staff capacity. After signing the Deed of Commitment with transition partners in 2005 that formalised the intent to transition the QH PHC services to Aboriginal community control, Gurriny started to expand its program and service range to include clinical health.

“(Gurriny started off as a Social Emotional Wellbeing Centre) then it graduated up into doing more primary health stuff” (ex-CHHHS, current Gurriny staff)

For 9 years following the signing of the Deed of Commitment (2005–2014) Gurriny built its services, workforce and organisational capacity. As the organisation grew and the official transition neared, the process became increasingly complex with regard to organisational operations as well as government expectations and requirements. In line with the increasing complexity, capacity building efforts focussed on Gurriny senior management, board member and staff. This was achieved through a continuous, cyclical process of planning, acting, monitoring and reporting. Plans outlined steps to address the core priority areas identified as key to transition; action was taken to implement the plans; actions were continuously monitored through the use of performance, status and readiness assessment reports; and the reported actions and development were then used to inform further planning as the cycle continued.

“all the way along, we did just keep chugging along, making the organisation better and smarter.” (Current Gurriny staff)

Prior to formal transition in June 2014, the Commonwealth government engaged a consultancy company, Bentley’s, to undertake an independent Organisational Capacity Review to demonstrate Gurriny capacity and ensure ongoing government support for transition. While this Organisational Capacity Review process was daunting, Gurriny embraced it as an opportunity for growth. In January 2015, Bentley’s completed their final assessment with Gurriny demonstrating progress across a number of organisational capacity pillars and particular strengths in community engagement, advocacy, service delivery, human resources and quality. Bentley’s commended Gurriny for its organisational growth and achievements over the 12-month assessment period.

“it is about capacity building… we looked at Gurriny internally and looked at all those pillars and thought, okay, this is what they want us to do… Let’s make sure that we tick the box that they want us to tick and make sure that Gurriny is ready in all those areas” (Current Gurriny staff)

Whilst Gurriny gained a great deal from the organisational capacity development process, the frequently stringent requirements and need to continuously demonstrate organisational and leadership capacity was a major challenge. Participants considered this requirement to stem from an underlying lack of trust from key stakeholders in Government and QH/CHHHS in Gurriny’s capacity. This lack of trust served to delay the transition and was one of its most significant barriers. While this and other barriers will be briefly addressed in this paper, they will be covered in greater detail in an upcoming paper on the barriers and enablers to transition.

### The transition process

**Fig. 1:**
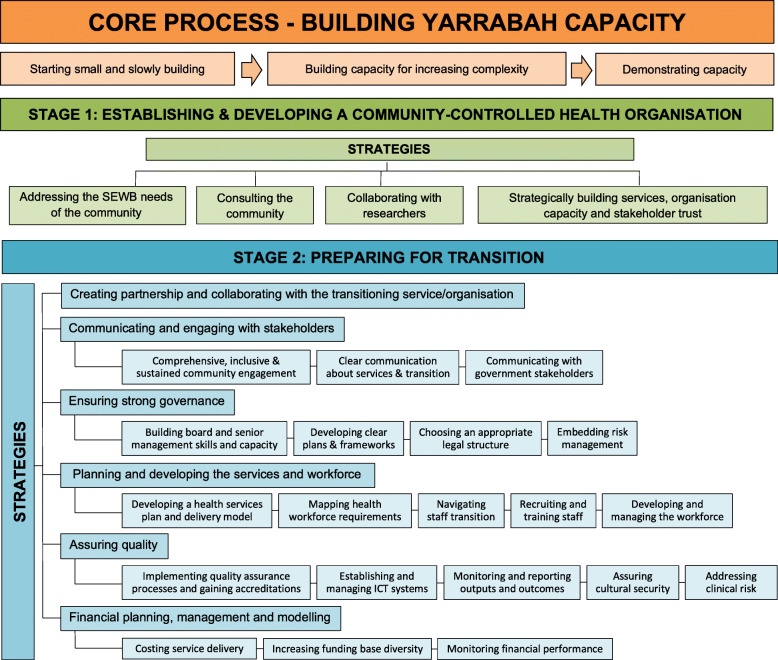
The process and strategies of transitioning to community control

As a lengthy process over several decades, the transition to community control of Yarrabah’s PHC involved two distinct stages (see Fig. 1 for an overview of the process and strategies of transition). The first stage began in 1986 and continued until 2004 and was centered on establishing and developing the organisational structure needed to take community control of PHC. The second stage started in 2005 with the signing of the Deed of Commitment and continued until 1 year after funding and service control was officially handed over to Gurriny on June 30, 2014. This second stage entailed intense organisational growth and development processes, including negotiations and collaboration between QH/CHHHS and Gurriny.

### Stage 1: establishing and developing a community-controlled health organisation

Four inter-related strategies contributed to developing Gurriny as an organisation during stage 1 of the transition. The first strategy was focused on addressing the SEWB needs of the community. Concerns around community health issues, particularly high levels of suicide that had occurred in Yarrabah, was one of the key driving forces behind community desire to gain control over PHC services.

Interconnected with the first strategy, the second strategy that emerged from the data was extensive community consultation which culminated in the 1998 Feasibility Study. The Study identified that intergenerational trauma related to stolen generations and loss of land, culture, and spirit associated with colonisation was largely responsible for the SEWB issues experienced in Yarrabah. The Study identified inadequate resources to respond to mental health issues in the community; a community desire for holistic health care which integrated SEWB care with bio-medical health care; and a strong community desire to take control of healthcare. It also recommended the establishment of a community-controlled health service focused on the service gap of SEWB services to build capacity and establish credibility, rather than competing with the bio-medical/clinical services provided by QH/CHHHS.

“…in the Yarrabah Feasibility Study… the community said… ‘We need to deliver our own Health Service but we’re not going to make a difference in our health unless we also address the issues that come from being a population of the stolen generation.’ So they made it really clear, ‘we want doctors, but don’t do it without addressing the social emotional wellbeing component ‘cause it’s just not going to work” (Gurriny staff member)

The next strategy was collaboration between the Yarrabah community and researchers. Soon after completion of the Feasibility Study, community leaders approached researchers to guide development of an evidence-based Centre of Excellence in SEWB. The Family Wellbeing (FWB) program, an Aboriginal developed SEWB program, was used to build local capacity in the Gurriny workforce and broader Yarrabah community and became a foundation of Gurriny’s SEWB services. Research evidence of the impacts of FWB helped demonstrate Gurriny’s capacity as a health service and secure further funding to employ staff and grow Gurriny’s workforce.

“we got our first… three year NHMRC grant to implement Family Wellbeing in Hopevale, Yarrabah, as well as Cairns. Out of the people we trained, we employed five… people… The very first core of Gurriny staff were actually research employed.” (Research partner)

The final strategy employed in the first stage of the transition was strategically building services, organisational capacity and stakeholder trust. Starting with minimal resources and a lack of confidence from key stakeholders in the capacity of Gurriny to successfully run a PHC, Yarrabah leaders took a strategic approach to growing the organisation and building trust in its services and capacity. With facilitation by researchers, the Family Wellbeing (FWB) program was delivered to Gurriny staff to strengthen local leadership and develop initial SEWB services, resulting in significant benefits for participants and increased community confidence in Gurriny’s capacity. Evaluations demonstrated the outcomes of FWB and helped to position Gurriny as a leader in the SEWB field, build government trust and secure government funding which was used to employ staff and grow programs. This strategy was ultimately aimed at getting commitment from government stakeholders to transition PHC services to community control.

“(Gurriny leader) was confident that if they could demonstrate capacity in the social and emotional wellbeing area, then the community would have more confidence in Gurriny’s ability to take on other services.” (Research partner)

### Stage 2: preparing for transition

The second stage of the transition process started in 2005 with the signing of the Deed of Commitment, and ended 1 year following the official handover of PHC services on 30th of June, 2014. This stage required thorough preparation, particularly in the final 2 years. The five strategies encompassed in stage 2 were: communicating & engaging with stakeholders; ensuring strong governance; planning and developing the services and workforce; assuring quality; and, financial planning, management and modelling.

#### Communicating & Engaging with stakeholders

Critical during the second stage of the transition was communication and engagement with key stakeholders. The primary stakeholders involved in the transition process were CHHHS and Gurriny staff, service users, and Yarrabah community members, organisations, groups and services. Community support for community control was a core requirement for the transition and was something that Gurriny continuously built through a process of comprehensive, inclusive and sustained engagement with the Yarrabah community. For example, Gurriny representatives regularly attended the meetings of local groups and committees, held public meetings and community information sessions, and shared information through community events as well as relevant websites and social media pages. The aim was to raise awareness of the transition, inform community members about progress and include them as decision makers in the process. Focus groups were facilitated to support input into service delivery decisions and Gurriny sought informal feedback to gauge community opinion regarding the transition.

“The main role I think that they had was one of us creating opportunities for them to say ‘no’, and being open to that... it was more about making sure that if somebody didn’t agree, we could hear about it.” (Gurriny staff)

One main goal of the communication and engagement strategy was to communicate clearly with the Yarrabah community about the service delivery changes that were occurring and outcomes being achieved as a result. Flyers and brochures which described the transition, programs and how to access Gurriny services were developed, Information on health data, outputs, and outcomes were communicated through newsletters, community information sessions and quarterly health snapshot summaries.

Significant effort went into engaging, communicating and planning with key government stakeholders. Between 2007 and 2013, three distinct strategy documents were developed to guide efforts towards communication, engagement and negotiation between Gurriny and QH/CHHHS. At the end of 2008, Gurriny established a local transition committee which met fortnightly to facilitate joint planning and development with CHHHS. Documents evaluated asserted that communication between Gurriny and CHHHS managers was aimed at building relationships, creating shared vision and progressing the transition as well as working through conflicts, negotiating an official transition date and joint planning and coordination of service delivery.

However, data from interviews and documents indicated that this was an area of ongoing contention. For example, from 2010 there was a shift in focus in transition planning from Aboriginal community control to service integration between CHHHS and Gurriny. This process was not Gurriny’s preferred option but occurred as a result of strong resistance from staff in the Yarrabah CHHHS service.

“the other thing that kept happening was we kept getting pushed towards a model where we all just worked together. So that it wasn’t about us being in control.” (Gurriny staff)

#### Ensuring strong governance

Gurriny’s community engagement processes and governance by a locally elected board of directors assured strong local Aboriginal governance of the organisation. However, in preparation for transition, Gurriny needed to ensure strong governance to meet the western managerial style of accountability expected by government. One way this was achieved was by enhancing governance skills and knowledge, roles and related behaviours and ethical expectations, and other management and leadership capabilities of Board Directors and Managers to deal with increased expectations, responsibilities and larger budgets. Director membership was expanded, hiring external consultants and utilising partnerships and alliances in the ACCHS sector.

Throughout much of the transition journey, various plans and frameworks were developed and implemented to guide aspects of PHC service governance. Business plans were iteratively reviewed and enhanced leading up to official handover to support the transition process and Gurriny’s ongoing organisational operations and development.

“my role was to facilitate the establishment of various planning documents around the transition. So we worked very closely with Apunipima Cape York Health Council… about setting up some of the frameworks… around service delivery, human resources and finance, funds pooling, legal, how to best prepare the health service around transitioning primary health care over to a community control model.” (ex-Gurriny staff)

Other strategies to ensure strong governance included deciding on an appropriate legal structure which suited the size and experience of the organisation; as well as the development of risk management plans and procedures.

#### Planning and developing the services & workforce

Another core strategic area in the transition process was the planning and development of Gurriny’s service delivery model and associated workforce. This began with the development of an enhanced PHC services plan and delivery model based on Yarrabah’s health service needs and options. The model focused on community-centred, culturally appropriate, comprehensive PHC including acute care, prevention and early intervention, addressing all stages of life, and including a range of healthcare programs to promote holistic health outcomes. The planned services were to be implemented by a community-based service delivery team and delivered through clinical, outreach, home visits and mobile services. The proposed model of care reflected best practice, evidence-based approaches and was aligned with policy directives such as the Council of Australian Governments’ “Closing the Gap” targets.

Once the health service organisational structure and service delivery model had been established, Gurriny then considered staffing requirements and mapped a workforce profile and position descriptions .

“the big task for us was to look at and plan how many staff we would need from doctors, Registered Nurses and Health Workers.” (ex-CHHHS, current Gurriny staff)

An important yet challenging aspect of building the required workforce was navigating the transition of CHHHS staff employment over to Gurriny. This unprecedented process involved complex industrial relations and workers’ rights legislation. Gurriny aimed to ensure that no CHHHS staff member lost their job in the transition process, however, had little control over decisions regarding how CHHHS employment issues would be managed. Only 3 months before the handover of services and funding CHHHS staff were offered a voluntary redundancy, however redundancy conditions stipulated that they could not apply for positions at Gurriny for 3 months. Therefore, Gurriny decided to fill positions on a temporary basis until the exclusion period was over. This was a complicated arrangement which had significant impacts for staffing Gurriny’s service delivery immediately following transition.

*“we worked out… we would only put on staff for three months to fill positions, to keep the wheels chugging along and then we would advertise the permanent positions and if a Queensland Health staff was interested in applying, they were welcome to apply.”* (Gurriny staff)

Gurriny implemented a staff induction program which aimed to ensure awareness of its policies and procedures and addressed potential differences in CHHHS and Gurriny operating frameworks. Several staff development processes and initiatives were also undertaken by Gurriny throughout the transition process including support for Aboriginal Health Workers to complete certificate four or a diploma in PHC on site and a weekly half-day of staff training and development. Lastly, a whole of staff change management workshop along with individual change management sessions were delivered to explain the transition to staff and educate them about the new service delivery model.

#### Assuring quality

Another core strategy area was quality assurance. This included the accreditation of Gurriny under the Royal Australian College of General Practitioners (RACGP), the International Organization for Standardization (ISO) 9001, and Australian General Practitioner Accreditation Limited (AGPAL). Other quality assurance processes implemented included: the completion of auditors’ training by staff members; staff recruitment training and onboarding focused on quality systems; and the recruitment of a manager with quality experience.

The development and application of effective information community technology (ICT) systems was also an area of focus for Gurriny. However, navigating issues related to the ownership, access, management, sharing, protection and storage of client data and information was an ongoing challenge throughout the transition.

Monitoring and reporting performance were needed to meet Gurriny’s accountability requirements to both government and the Yarrabah community, as well as to address Gurriny’s goals of evaluating the health reform process and continuous quality improvement. Service delivery and clinical performance were evaluated against national Key Performance Indicators.

Gurriny also needed to demonstrate to governments, staff and community members that its services were culturally appropriate and secure. Efforts to ensure the cultural security of the organisation included: reflecting community cultural values in the model of care; having multidisciplinary teams involving local Aboriginal Health Workers provide holistic health care; educating staff about Gurriny’s model of care to increase awareness of organisational culture; and providing cultural awareness training with all staff.

Finally, to address clinical risk systems were established to monitor and capture critical incidents. This included setting up monthly clinical incident meetings and putting in place an Asset Management Register and Safety Checks for equipment.

#### Financial planning, management and modelling

The final core strategy area required for a successful transition was financial planning, management and modelling. Central to this was the costing Gurriny’s service delivery, however different approaches were taken towards this. Early in the transition process, external consultants were commissioned by the Queensland Aboriginal and Islander Health Council (QAIHC) to calculate the level of public funding that would be required to implement the transition to community control while supporting both increased service utilisation and improved health outcomes. The resultant Eagar and Gordon (2008) report recommended an equity-based funds pooling approach which included core primary healthcare funding at double the rate of national average expenditure on non-Indigenous Australians, as well as funds from the Pharmaceuticals Benefits Scheme and Medicare Benefits Scheme.

Much later in the transition, CHHHS hired an external auditor to cost the delivery of the PHC services which were to be transitioned to determine the amount of funding allocation. In this instance there was a drive to make the transition cost neutral, therefore only the minimal level of services provided by CHHHS were assessed, without accounting for differences in Gurriny’s model of care or service delivery improvements. This was seen by both Gurriny and QH participants in this evaluation as being an inappropriate costing method and inconsistent with the kind of equity-based funding approach recommended in the Eagar and Gordon report.

Throughout the transition process, Gurriny also increased the diversity of its funding streams, particularly through increased monthly Medicare revenue. Other efforts to diversify Gurriny’s funding base included: leveraging off universities and not-for-profit organisations; working with external agencies to access philanthropic funds; and exploring the opportunity of using Medicare revenue to invest in capital equipment for Gurriny’s future growth and expansion.

## Discussion

Since their inception in the 1970s to the present day, ACCHSs have played a critical role in the efforts towards achieving healthcare equity for Aboriginal people in Australia [6, 9, 14]. Beyond addressing key PHC barriers related to the affordability, availability, cultural acceptability and appropriateness of health care to health needs, ACCHSs also provide an important avenue for community empowerment and leadership development in Aboriginal communities [6, 10]. Having community control of essential services such as PHC continues to be a strong aspiration of Aboriginal communities in their ongoing pursuit of self-determination [7, 11].

Although the literature base on the development of Indigenous community controlled PHC globally has grown in the last couple of decades, very few evaluations of transitions of government PHC to community control have been undertaken, with none reported in the peer-reviewed literature (Dwyer, et al. [10]; Lavoie, et al. 2006). Furthermore, whilst evaluated experiences in the Australian context provide insight into challenges experienced through attempted transitions (Dwyer, et al., [10]), there seems to be a dearth of literature on the process and strategies involved in successful transitions.

Gurriny’s experience of transitioning to community control tells a complex story of both challenge and triumph. Gurriny achieved transition of PHC services in Yarrabah to full community control, suggesting that the transition process was successful in terms of achieving its primary goal. It is also evident that through the process of transitioning to community control, Gurriny as an organisation accomplished significant growth in organisational and leadership capacity. For some Gurriny staff, the whole organisational development process, while challenging and daunting, was a positive experience that helped Gurriny to become stronger as an organisation.

Yet in the process of achieving the success of transition, Gurriny had to navigate complex organisational and political conditions and contexts that often worked against the Gurriny and Yarrabah vision of community control of PHC [23]. The operation of a comprehensive PHC service entails responsibility both to community; to whom ACCHSs have a commitment to provide quality, culturally acceptable, accessible health care which is responsive to the health needs of community and community wishes; and to government, who need to be assured of the quality, performance and cost effectiveness of the services they fund. The balancing of these different responsibilities is one of the most challenging yet critical roles of ACCHSs [24].

These distinct and sometimes conflicting responsibilities are reflected in the complex strategies implemented by Gurriny to achieve transition. Efforts to meet government requirements of capacity building in order to be approved for the transition are seen in the areas of governance, quality, workforce and service development and finance. Competence and capacity frameworks such as the Bentley’s Organisational Capacity Review and others implemented in other ACCHSs [10], are very stringent and are beyond the performance requirements of government PHC services [10]. Despite this, Gurriny, like other ACCHSs [10], succeeded in meeting these rigorous requirements, demonstrating the organisation’s strength and capacity. A lesson here for other communities or ACCHSs who are considering transition of PHC services to community control is to adopt a growth mindset and use the process of meeting government requirements as an opportunity to strengthen the organisation and improve capacity.

There are also many strategies which were more focused on meeting responsibilities to community. This is particularly seen in Gurriny’s ongoing community consultation process and community governance model, as well as in the comprehensive, holistic and culturally grounded model of care and strong local Aboriginal workforce. Research has found that these elements of ACCHS’s which make them so unique are also the characteristics that are so valued by Aboriginal patients. Principally, these are the holistic, comprehensive services which are responsive to community health need and are culturally safe [25].

However, as this evaluation shows, much of Gurriny’s process and strategies in transitioning to community control reflect mainstream organisational change and development processes. Considering the lack of research that exists on specific organisational change processes for Indigenous organisations [12, 23] ACCHS’s may need to look to the literature on managing organisational transitions in mainstream organisational contexts to gain insights [26–29].

Another learning from Gurriny’s experience of transition is that the journey to achieving community control of PHC takes time; it is important that communities have realistic expectations about how long the process can take. Gurriny’s journey took approximately 28 years from its first visioning by community leaders in the late 80s. However, the first 15 years of Gurriny’s journey was focused on establishing a community-controlled organisation. Even from 2005 when the Deed of Commitment was signed by all partners, the official transition did not happen for another 9 years, despite the expected transition period outlined in the Deed of Commitment of three to 5 years. Other successful and attempted transitions in Australia have similarly taken longer than expected [10]. Indeed, it has been recognised that the process of achieving community control in Australia has been hindered by unrealistic timelines [15]. Therefore it is critical that there is commitment to establishing clear dates and adherence by transition partners to agreed timeframes.

### Limitations

A strength of our evaluation is the use of multiple sources of data and methods to triangulate findings. The historical documents and interviews provide rich and comprehensive sources of data about the Gurriny processes that unfolded to achieve the transition to community control, and people’s retrospective perceptions of the process. However, there were a number of limitations to the project.

One major limitation was the lack of data from QH/CHHS about their experience of the transition. Although 7/14 participants were either current or ex QH staff (including three current Gurriny staff who previously worked for QH), there was a high refusal rate from previous and current QH staff members who were invited to participate (see Table 1). Furthermore, for the document analysis, similar documents from QH could not be accessed due to limitations of the project’s ethics approval. Acquiring additional ethical approval to access QH documents was beyond the time limits of the project. Therefore, the only QH/CHHHS reports or documents included for analysis were those Gurriny could provide. This limitation meant that the focus of the process evaluation was driven by Gurriny and may have overlooked areas of importance to Queensland Health.

## Conclusion

Gurriny’s journey of transitioning to community control of PHC in Yarrabah entailed a 28-year process of organisational and leadership capacity development. This process was required to meet government expectations and to ensure readiness to undertake the complex task of operating a large, comprehensive PHC service. The core strategy areas outlined in this paper are indicative of the capacity development activities required to achieve transition and provide a framework that may be useful for other ACCHSs and governments. The process of achieving community control has resulted in significant positive impacts for Yarrabah. However, communities who are considering similar transitions need to be aware of the complexities and responsibilities involved. They also need to have realistic expectations regarding how long transition can take, and maintain patience and persistence for what can be a long and challenging journey. Government stakeholders can support future transitions by providing clear information about expectations and requirements early on, helping to resource ACCHSs capacity strengthening processes, and having trust in Aboriginal governance. There is also a pressing need address resistance to community control among government PHC service providers and the underpinning systematic racism and mistrust which can lead to significant delays and act as a major barrier to transition.

## Data Availability

The datasets generated and analysed during the current study are not publicly available due to participant confidentiality but are available from the corresponding author on reasonable request.

## Abbreviations

ACCHS: Aboriginal Community Controlled Health Service
AGPAL: Australian General Practitioner Accreditation Limited
CIHER: Centre for Indigenous Health Equity Research
CHHHS: Cairns and Hinterland Hospital Health Service
CQU: Central Queensland University
FWB: Family Wellbeing
ICT: Information communication technology
ISO: International Organisation for Standardisation
NHMRC: National Health and Medical Research Council
ORIC: Office of the Registrar of Indigenous Corporations
PHC: Primary health care
QAIHC: Queensland Aboriginal and Islander Health Council
QH: Queensland Health
RACGP: Royal Australian College of General Practitioners
SEWB: Social and emotional wellbeing

## Footnotes

1 The terms ‘Aboriginal’ and ‘Torres Strait Islander’ refer to the many peoples and language groups who were living in Australia before European settlement, and who have experienced colonialism for more than 200 years. We use the term ‘Aboriginal’ in this paper to represent both Aboriginal and Torres Strait Islander peoples unless specifically referring to Torres Strait Islander peoples. We use the term ‘Indigenous’ for the Aboriginal peoples of other countries.

2 Sorry business is the term that is used by Aboriginal people in Australia to refer to a period of cultural practices and protocols associated with death or other experiences of grief or loss.
